# Expression and prongostic impact of galectin-7 in human lung cancer

**DOI:** 10.1097/MD.0000000000039911

**Published:** 2024-10-04

**Authors:** Anna Tirilomi, Petros Tirilomis, Omar Elakad, Sha Yao, Marc Hinterthaner, Bernhard C. Danner, Philipp Ströbel, Theodor Tirilomis, Hanibal Bohnenberger, Alexander von Hammerstein-Equord

**Affiliations:** a Department of Cardio-Thoracic and Vascular Surgery, University Medical Center, Göttingen, Germany; b Clinic for Cardiology & Pneumology, University Medical Center, Göttingen, Germany; c Institute of Pathology, University Medical Center, Göttingen, Germany.

**Keywords:** galectin-7, human lung cancer

## Abstract

Malignant tumors of the lung are the leading cancers worldwide. Prognostic biomarkers continue to be investigated for the detection and stratification of lung cancer for clinical use. In breast cancer cells and in lymphomas, the overexpression of galectin-7 led to increased metastasis. In lung cancer, squamous cell carcinoma, galectin-7 was also identified as a factor promoting metastasis. In this study, we investigated the expression of galectin-7 in relation to clinicopathological features and overall survival in patients with different types of lung cancer. By immunohistochemistry, expression of galectin-7 was analyzed in 308 cases of lung cancer; 108 cases of adenocarcinoma, 193 cases of squamous cell lung carcinoma and 7 cases of small cell lung cancer (SCLC) and correlated with clinicopathological characteristics as well as patients’ overall survival. Immunohistochemical detection of galectin-7 expression was most evident in squamous cell lung carcinoma (36.27%), followed by adenocarcinoma (5.55%). Negative expression of galectin-7 was found in all patients with SCLC. No significant correlation was found in patients with squamous cell lung carcinoma. Within the adenocarcinoma and squamous cell lung carcinoma subgroups, there were statistically significant correlations between the expression of galectin-7 and some clinicopathologic features of the patients. In our study, we were able to show that galectin-7 could serve as a new prognostic biomarker and is also a potential new drug target in non-small cell lung cancer.

## 1. Introduction

Lung cancer is worldwide the leading cause of cancer-associated mortality.^[1]^ Lung cancer is divided into small cell lung cancer (SCLC) and non-SCLC (NSCLC). The latter is further subdivided into adenocarcinoma (AC) and squamous cell lung carcinoma (SQCLC).^[2]^ The treatment of lung cancer depends on the TNM classification and Union for international cancer control, UICC stage.^[3]^ Surgery is the treatment of choice for patients with stage I or II lung cancer if there are no contraindications. The treatment claim is curative.^[4–6]^ Patients with stage III NSCLC are rarely treated surgically. Most patients receive radiotherapy and/or chemotherapy.^[7]^ In addition, lung cancer can be divided into many molecularly heterogeneous subgroups that are constantly evolving and potentially actionable. New strategies for molecular diagnostics and targeted therapies for lung cancer are being researched in order to detect and treat them.^[8–10]^

The protein galectin-7 is in humans encoded by the LGALS7 gene.^[11,12]^ Galectin-7 was originally considered a marker for the differentiation status of keratinocytes.^[11,12]^ Apoptotic keratinocytes expressed a high amount of galectin-7.^[13]^ In colon cancer cells, galectin-7 has been shown to act as an apoptosis regulator.^[14]^ Furthermore, it was shown that galectin-7 makes tumor cells more susceptible to apoptotic stimuli.^[15,16]^ On the other hand, the growth of neuroblastoma cells was reduced by extracellular binding of galectin-7 to cell surface receptors. There was no evidence of classical apoptosis.^[17]^

In breast cancer cells, overexpression of galectin-7 led to a significant increase in lung metastases and osteolytic lesions. Galectin-7 is therefore being further investigated as a potential target for the specific detection and therapeutic inhibition of metastatic breast cancer.^[18]^ In patients with breast cancer, galectin-7 was investigated as a prognostic marker and found to be associated with disease-free survival.^[19]^

In addition to breast cancer, it has also been shown in lymphomas that overexpression of galectin-7 leads to increased metastasis.^[20,21]^

In melanoma cells, the expression of galectin-7 was shown on primary tumors and lung metastases. The overexpression of galectin-7 increased the resistance of melanoma cells to apoptosis, but the overexpression had no effect on tumor growth.^[22]^

In a mouse model of lung cancer with squamous cell carcinoma cells, galectin-7 was shown to be a key mediator of metastasis in conjunction with immunosuppression. Galectin-7 was identified as a metastasis-promoting factor.^[23]^

In this study, the expression of galectin-7 was investigated in patients with non-small cell (adenocarcinoma (AC) and squamous cell carcinoma (SQCLC)) and SCLC in relation to clinicopathological characteristics and overall survival.

## 2. Material and methods

Tissue samples of lung cancer from patients who underwent surgery at the Department of Thoracic and Cardiovascular Surgery, University Medical Center Göttingen, Germany were analyzed for galectin-7 expression. All patients signed an informed consent. In accordance with the Declaration of Helsinki (October 2013 version) and institutional, state and federal guidelines and after approval by the ethics committee of the University Medical Center Göttingen (#1-2-08), the study was conducted. Inclusion criteria were age > 18 years and presence of histology of AC, SQCLC or SCLC. Exclusion criteria were a different histology, patients with inoperable tumor or with neoadjuvant treatment.

### 2.1. Immunohistochemical staining

The 2-mm tissue sections were incubated in EnVision Flex Target Retrieval Solution at low PH (Dako/agilent, Santa Clara, CA), followed by incubation with the primary antibody against galectin-7 (Novus Bio, #NPB2-33995, 1:500) for 20 minutes at room temperature. Visualization was performed with a polymeric secondary antibody coupled to horseradish peroxidase (EnVision Flex+, Dako) and DAB (Dako). Prior to the light microscopy analysis of the tissue samples, counterstaining with Meyer’s hematoxylin was performed. The findings were classified according to the intensity of the staining into negative, weak expression and strong expression.

### 2.2. Statistical analysis

Data were collected using Microsoft Excel 2019 and statistical analysis was performed using GraphPad Prism (version 7.00 for Windows, GraphPad Software, La Jolla, CA, www.graphpad.com). The correlation between clinicopathological features and protein expression of galectin-7 was analyzed using Chi-Square test and Student *t* test. The Kaplan–Meier estimator was used to investigate patient survival in relation to galectin-7 expression. Differences were calculated using the Mantel-Cox log-rank test. *P*-values < .05 were considered statistically significant.

## 3. Results

### 3.1. Patient characteristics

A total of 308 patients (226 men, 82 women) were included in this study. The average age of the patients was 66 years (range 34–85 years), with two/thirds of the patients being older than 60 years. Histologically, non-SCLC (NSCLC) was the leading type. Squamous cell carcinoma (SQCLC) was detectable in 193 patients and adenocarcinoma in 108 patients. Only 7 patients had small cell lung carcinoma (SCLC). Lymph node metastasis could be ruled out in over 50% of patients. R0 resection status was achieved in around 90% of patients. Patients in clinical stage III (IIIa n = 66, IIIb n = 8) and stage IV (n = 6) received neoadjuvant treatment prior to surgery. Surgery of patients in stage IV was performed with palliative intention. Table 1 and Supplementary Table 1, http://links.lww.com/MD/N665 show the detailed data on patient characteristics, clinical status and degree of tumor differentiation.

**Table 1:** Patient characteristics according to histopathology.

	Cases	AC	SQCLC	SCLC
Total *n*	308	108	193	7
Age median (range)	66 (34–85)	65 (34–81)	66 (39–85)	67 (59–74)
Gender				
Male	226	66	153	7
Female	82	42	40	0
Age				
≤60	94	41	51	2
>60	213	66	142	5
Degree of differentiation				
I + II	219	77	147	0
III	81	29	45	7
Lymph node metastasis				
No	174	61	108	5
Yes	119	40	78	1
Clinical stage				
I + II	220	80	138	2
III + IV	80	26	54	0
Resection status				
R0	273	96	173	4
R1 + 2	26	6	18	2

AC = adenocarcinoma, SCLC = small cell lung cancer, SQCLC = squamous cell lung cancer.

### 3.2. Impact of galectin-7 expression and survival

**Figure 1.**
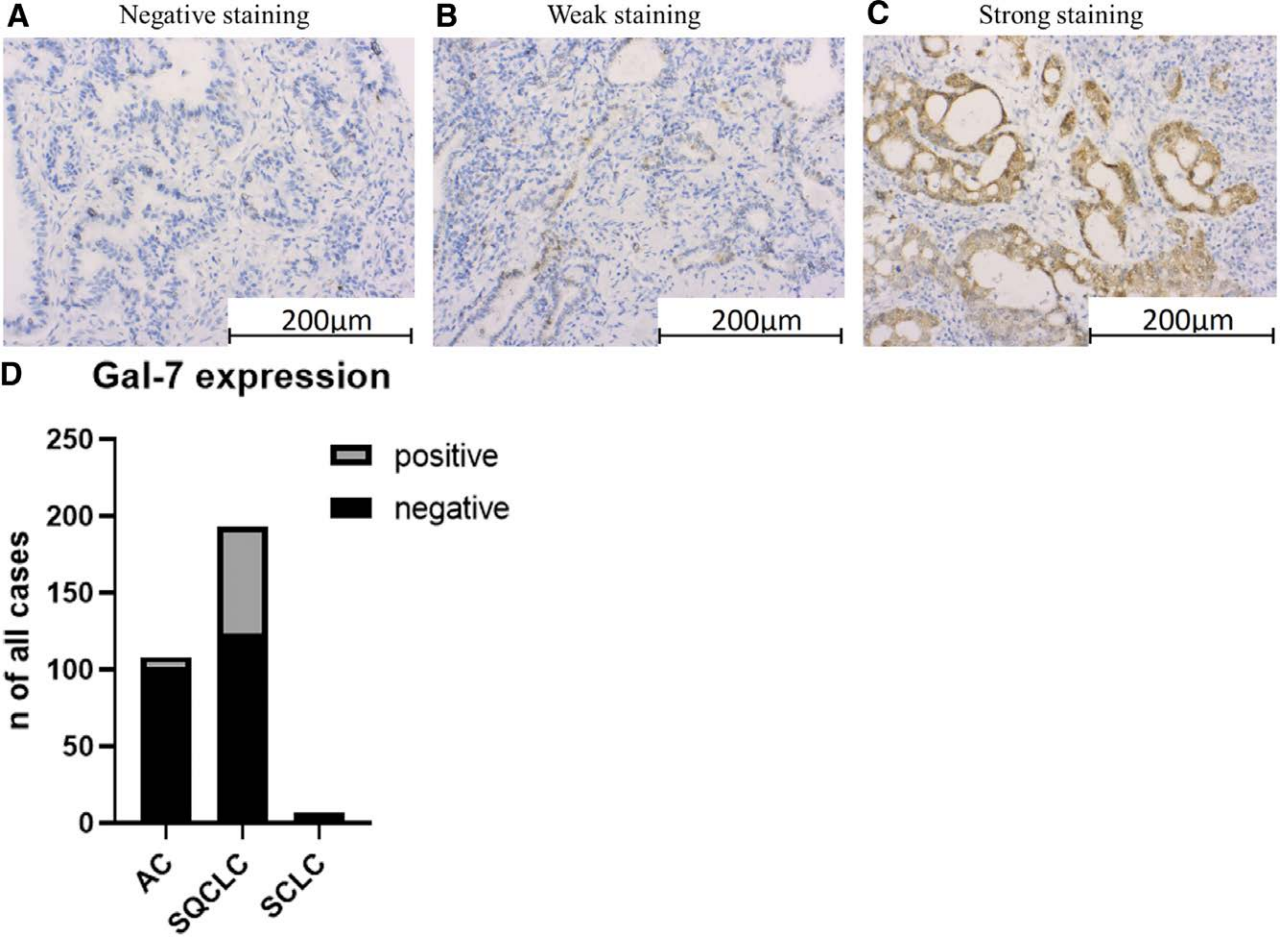
Representative immunohistochemical staining with negative (A), weak (B) or strong (C) immunostaining of galectin-7 in lung cancer samples. Scale bar: 200 µm. (D) galectin-7 expression sorted by entity and categorized as positive and negative expression.

In the immunohistochemical study, strong or weak expression of galectin-7 was most evident in 70 patients (36.27%) with SQCLC, followed by 6 patients (5.55%) with AC. Negative expression for galectin-7 was found in all patients with SCLC (100%), followed by 102 patients (94.44%) with AC and 123 patients (63.73%) with SQCLC (Fig. 1).

**Figure 2.**
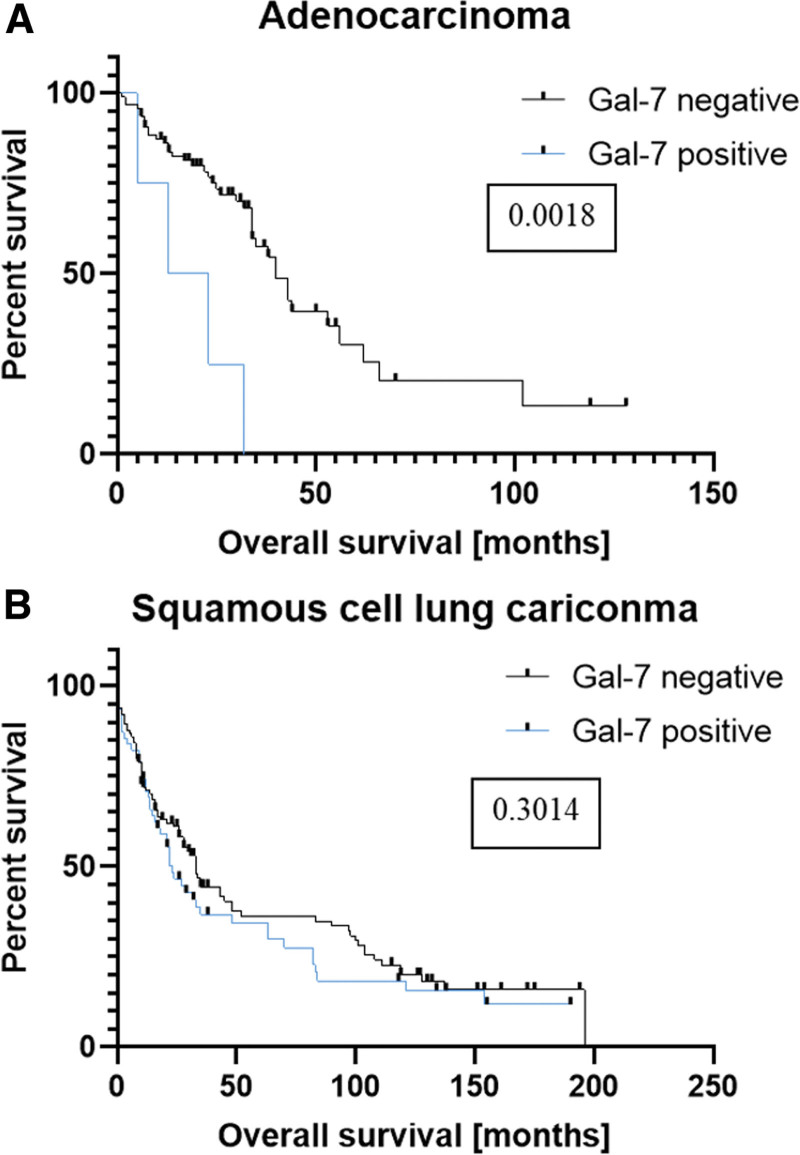
Kaplan–Meier analysis of overall survival in patients with AC (A) and SQCLC (B). The *P*-value is from a log-rank test.

The correlation between galectin-7 expression and overall survival of patients with AC and SQCLC was demonstrated using the Kaplan–Meyer-Estimation. The median follow-up time was 24 months (range 6–128). No significant correlation was found in patients with SQCLC (Fig. 2).

### 3.3. Protein expression of galectin-7 in lung cancer patients and correlation with clinicopathological characteristics

In patients with AC, a significant correlation was found between the expression of galectin-7 and the age of the patients (Table 2). In addition, a significant correlation between the expression of galectin-7 and the degree of differentiation was found in patients with SQCLC (Table 3).

**Table 2:** Galectin-7 expression in adenocarcinoma sorted by clinical features.

		Galectin-7		
	Cases	−	+	*P*-value
Gender				
Male	66	63	4	
Female	42	39	3	.808
Age				
≤60	41	41	0	
>60	66	60	6	.047
Degree of differentiation				
I + II	77	73	4	
III	29	27	2	.735
Lymph node metastasis				
No	61	59	2	
Yes	40	36	4	.162
Clinical stage				
I + II	80	77	3	
III + IV	26	23	3	.135
Resection status				
R0	96	91	5	
R1 + 2	6	5	1	.247

*P*-values are calculated according to Chi-square test.

**Table 3:** Galectin-7 expression in squamous cell lung carcinoma sorted by clinical features.

		Galectin-7		
	Cases	−	+	*p*-value
Gender				
Male	153	95	58	
Female	40	28	12	.354
Age				
≤60	51	31	20	
>60	142	92	50	.610
Degree of differentiation				
I + II	147	86	61	
III	45	37	8	.004
Lymph node metastasis				
No	108	73	47	
Yes	78	35	31	.302
Clinical stage				
I + II	138	90	47	
III + IV	54	33	21	.135
Resection status				
R0	173	110	63	
R1 + 2	18	12	6	.795

*P*-values are calculated according to Chi-square test.

## 4. Discussion

Lung cancer continues to be the most common cause of death from cancer worldwide.^[24]^ Patients are treated according to the TNM classification and the UICC stage of the cancer. Surgical resection is the treatment of choice in stages I and II of NSCLC. From stage II onwards, adjuvant therapy increases.^[25,26]^ It has recently been shown that lung cancer can be divided into molecularly heterogeneous subgroups and that individualized therapy is important.^[27]^

In this study, immunohistochemical detection of galectin-7 expression was most evident in SQCLC, followed by AC. Negative expression of galectin-7 was found in all patients with SCLC. Statistically significant correlations between the expression of galectin-7 and some clinicopathologic features of the patients were found in AC and SQCLC.

Galectin-7 has been shown to be an important mediator in various tumor entities. In breast cancer, galectin-7 promotes metastatic potential and resistance to apoptosis.^[28]^ Overexpression of galectin-7 has been found in squamous cell carcinoma of the esophagus.^[29]^ In melanoma, galectin-7 was detected both in the primary focus and in the lung metastasis.^[22]^ It has also been shown that galectin-7 promotes bone and lung metastasis of breast cancer.^[18]^ It was found that galectin-7 is elevated in lung cancer patients with a squamous cell histology.^[30]^

In squamous cell carcinoma, the expression of galectin-7 has been shown to be induced during tumorigenesis, particularly in the microenvironment of immunosuppression. During tumor progression, galectin-7 is released extracellularly. By deleting galectin-7, metastasis could be suppressed without affecting the growth of the primary tumor. Therefore, galectin-7 is considered a mediator of tumor metastasis and could be a potential target of cancer immunotherapy.^[23]^

In our study, we were able to show that galectin-7 can serve as a prognostic biomarker, particularly in patients with non-SCLC, and may be a potential new drug target. The mechanistic role of galectin-7 in the tumorigenesis of non-SCLC and the importance of galectin-7 as a therapeutic target should be further investigated.

Due to the design of a single center, the results of the current study are limited. A multicenter study with a larger patient cohort is therefore proposed.

## Acknowledgments

The authors acknowledge support by the Open Access Publication Funds of Göttingen University. The authors thank Jennifer Appelhans for her technical support.

## Author contributions

**Conceptualization:** Philipp Ströbel, Hanibal Bohnenberger.

**Data curation:** Anna Tirilomi, Petros Tirilomis, Omar Elakad, Sha Yao, Marc Hinterthaner, Hanibal Bohnenberger, Alexander von Hammerstein-Equord.

**Formal analysis:** Anna Tirilomi, Petros Tirilomis, Omar Elakad, Sha Yao, Theodor Tirilomis, Hanibal Bohnenberger.

**Funding acquisition:** Sha Yao, Philipp Ströbel.

**Investigation:** Omar Elakad, Marc Hinterthaner, Philipp Ströbel, Hanibal Bohnenberger.

**Methodology:** Omar Elakad, Sha Yao, Marc Hinterthaner, Philipp Ströbel, Hanibal Bohnenberger.

**Project administration:** Philipp Ströbel, Hanibal Bohnenberger, Alexander von Hammerstein-Equord.

**Resources:** Philipp Ströbel.

**Software:** Omar Elakad, Philipp Ströbel.

**Supervision:** Bernhard C. Danner, Philipp Ströbel, Hanibal Bohnenberger, Alexander von Hammerstein-Equord.

**Validation:** Anna Tirilomi, Theodor Tirilomis, Bernhard C. Danner, Philipp Ströbel, Hanibal Bohnenberger.

**Visualization:** Theodor Tirilomis, Bernhard C. Danner, Philipp Ströbel, Hanibal Bohnenberger.

**Writing – original draft:** Anna Tirilomi.

**Writing – review & editing:** Alexander von Hammerstein-Equord.

## Supplementary Material



## References

[1] Fitzmaurice C Abate D Abbasi N. Global Burden of Disease Cancer Collaboration. Global, regional, and national cancer incidence, mortality, years of life lost, years lived with disability, and disability-adjusted life-years for 29 cancer groups, 1990 to 2017: a systematic analysis for the global burden of disease study. JAMA Oncol. 2019;5:1749–68. 31560378 10.1001/jamaoncol.2019.2996 PMC6777271

[2] Travis WD Brambilla E Nicholson AG. WHO Panel. The 2015 World Health Organization classification of lung tumors: impact of genetic, clinical and radiologic advances since the 2004 classification. J Thorac Oncol. 2015;10:1243–60. 26291008 10.1097/JTO.0000000000000630

[3] Goldstraw P Crowley J Chansky K. International Association for the Study of Lung Cancer International Staging Committee. The IASLC lung cancer staging project: proposals for the revision of the TNM stage groupings in the forthcoming (seventh) edition of the TNM classification of malignant tumours. J Thorac Oncol. 2007;2:706–14. 17762336 10.1097/JTO.0b013e31812f3c1a

[4] Saji H Okada M Tsuboi M. West Japan Oncology Group and Japan Clinical Oncology Group. Segmentectomy versus lobectomy in small-sized peripheral non-small-cell lung cancer (JCOG0802/WJOG4607L): a multicentre, open-label, phase 3, randomised, controlled, non-inferiority trial. Lancet. 2022;399:1607–17. 35461558 10.1016/S0140-6736(21)02333-3

[5] Wen SW Han L Lv HL. A propensity-matched analysis of outcomes of patients with clinical stage I non-small cell lung cancer treated surgically or with stereotactic radiotherapy a meta-analysis. J Invest Surg. 2019;32:27–34. 28985095 10.1080/08941939.2017.1370519

[6] Cao C Wang D Chung C. A systematic review and meta-analysis of stereotactic body radiation therapy versus surgery for patients with non-small cell lung cancer. J Thorac Cardiovasc Surg. 2019;157:362–73.e8. 30482524 10.1016/j.jtcvs.2018.08.075 PMC6582640

[7] Eberhardt WE Pöttgen C Gauler TC. Phase III study of surgery versus definitive concurrent chemoradiotherapy boost in patients with resectable stage IIIA(N2) and selected IIIB non-small-cell lung cancer after induction chemotherapy and concurrent chemoradiotherapy (ESPATUE) J. Clin. Oncol. 2015;33:4194–201. 10.1200/JCO.2015.62.6812 26527789

[8] Wu YL Tsuboi M He J. ADAURA Investigators. Osimertinib in resected EGFR-mutated non-small-cell lung cancer. N Engl J Med. 2020;383:1711–23. 32955177 10.1056/NEJMoa2027071

[9] D’Angelo SP Janjigian YY Ahye N. Distinct clinical course of EGFR-mutant resected lung cancers: results of testing of 1118 surgical specimens and effects of adjuvant gefitinib and erlotinib. J Thorac Oncol. 2012;7:1815–22. 23154553 10.1097/JTO.0b013e31826bb7b2

[10] Jonna S Subramaniam DS. Molecular diagnostics and targeted therapies in non-small cell lung cancer (NSCLC): an update. Discov Med. 2019;27:167–70. 31095926

[11] Madsen P Rasmussen HH Flint T. Cloning, expression, and chromosome mapping of human galectin-7. J Biol Chem. 1995;270:5823–9. 7534301 10.1074/jbc.270.11.5823

[12] Magnaldo T Bernerd F Darmon M. Galectin-7, a human 14 kDa S-lectin, specifically expressed in keratinocytes and sensitive to retinoic acid. Dev Biol. 1995;168:259–71. 7729568 10.1006/dbio.1995.1078

[13] Bernerd F Sarasin A Magnaldo T. Galectin-7 overexpression is associated with the apoptotic process in UVB-induced sunburn keratinocytes. Proc Natl Acad Sci USA. 1999;96:11329–34. 10500176 10.1073/pnas.96.20.11329 PMC18033

[14] Polyak K Xia Y Zweier JL Kinzler KW Vogelstein B. A model for p53-induced apoptosis. Nature. 1997;389:300–5. 9305847 10.1038/38525

[15] Kuwabara I Kuwabara Y Yang RY. Galectin-7 (PIG1) exhibits pro-apoptotic function through JNK activation and mitochondrial cytochrome c release. J Biol Chem. 2002;277:3487–97. 11706006 10.1074/jbc.M109360200

[16] Ueda S Kuwabara I Liu FT. Suppression of tumor growth by galectin-7 gene transfer. Cancer Res. 2004;64:5672–6. 15313906 10.1158/0008-5472.CAN-04-0985

[17] Kopitz J Andre S von Reitzenstein C. Homodimeric galectin-7 (p53-induced gene 1) is a negative growth regulator for human neuroblastoma cells. Oncogene. 2003;22:6277–88. 13679866 10.1038/sj.onc.1206631

[18] Demers M Rose AA Grosset AA. Overexpression of galectin-7, a myoepithelial cell marker, enhances spontaneous metastasis of breast cancer cells. Am J Pathol. 2010;176:3023–31. 20382700 10.2353/ajpath.2010.090876 PMC2877862

[19] Ramos-Martínez JC Altamirano-Gómez G Ramos-Martínez I. Prognostic value of galectin expression in patients with breast cancer: systematic review and meta-analysis. Clin Breast Cancer. 2022;22:399–409. 35058144 10.1016/j.clbc.2021.12.011

[20] Demers M Magnaldo T St-Pierre Y. A novel function for galectin-7: promoting tumorigenesis by up-regulating MMP-9 gene expression. Cancer Res. 2005;65:5205–10. 15958565 10.1158/0008-5472.CAN-05-0134

[21] Demers M Biron-Pain K Hébert J Lamarre A Magnaldo T St-Pierre Y. Galectin-7 in lymphoma: elevated expression in human lymphoid malignancies and decreased lymphoma dissemination by antisense strategies in experimental model. Cancer Res. 2007;67:2824–9. 17363605 10.1158/0008-5472.CAN-06-3891

[22] Biron-Pain K Grosset A-A Poirier F Gaboury L St-Pierre Y. Expression and functions of galectin-7 in human and murine melanomas. PLoS One. 2013;8:e63307. 23658821 10.1371/journal.pone.0063307 PMC3643947

[23] An J Nagaki Y Motoyama S. Identification of Galectin-7 as a crucial metastatic enhancer of squamous cell carcinoma associated with immunosuppression. Oncogene. 2022;41:5319–30. 36335283 10.1038/s41388-022-02525-1

[24] Sung H Ferlay J Siegel R. Global cancer statistics 2020: GLOBOCAN estimates of incidence and mortality worldwide for 36 cancers in 185 countries. CA Cancer J Clin. 2021;71:209–49. 33538338 10.3322/caac.21660

[25] Miller KD Nogueira L Devasia T. Cancer treatment and survivorship statistics, 2022. CA Cancer J Clin. 2022;72:409–36. 35736631 10.3322/caac.21731

[26] Mallin K Browner A Palis B. Incident cases captured in the National Cancer Database compared with those in U.S. population based central cancer registries in 2012-2014. Ann Surg Oncol. 2019;26:1604–12. 30737668 10.1245/s10434-019-07213-1

[27] Cordero RR Devine WP. Targeted therapy and checkpoint immunotherapy in lung cancer. Surg. Path. 2020;13:17–33. 10.1016/j.path.2019.11.002 32005431

[28] Campion CG Labrie M Lavoie G St-Pierre Y. Expression of galectin-7 is induced in breast cancer cells by mutant p53. PLoS One. 2013;8:e72468. 23967302 10.1371/journal.pone.0072468 PMC3743813

[29] Zhu X Ding M Yu ML Feng M-X Tan L-J Zhao F-K. Identification of galectin-7 as a potential biomarker for esophageal squamous cell carcinoma by proteomic analysis. BMC Cancer. 2010;10:290. 20546628 10.1186/1471-2407-10-290 PMC3087317

[30] Blair BB Funkhouser AT Goodwin JL. Increased circulating levels of galectin proteins in patients with breast, colon, and lung cancer. Cancers. 2021;13:4819. 34638303 10.3390/cancers13194819 PMC8508020

